# *Trypanosoma cruzi* (Chagas, 1909) transmission among captive wild mammals, triatomines and free-living opossums from surrounding areas in the São Paulo Zoological Park, Brazil

**DOI:** 10.1371/journal.pntd.0013055

**Published:** 2025-04-28

**Authors:** Suelen Sanches Ferreira, Marina Galvão Bueno, Carolina V. C. Nery, Fabrício B. Rassi, Cauê Monticelli, Carolina R. F. Chagas, Irys H. L. Gonzalez, Samanta Cristina das Chagas Xavier, Cristiane Varella Lisboa, Patrícia Locosque Ramos, André Luiz Rodrigues Roque

**Affiliations:** 1 Laboratório de Biologia de Tripanosomatídeos, Instituto Oswaldo Cruz, Rio de Janeiro, Rio de Janeiro, Brasil,; 2 Laboratório de Virologia Comparada e Ambiental, Instituto Oswaldo Cruz, Rio de Janeiro, Rio de Janeiro, Brasil,; 3 Reserva Paulista Administradora de Parques, São Paulo, São Paulo, Brasil; 4 Coordenadoria de Fauna Silvestre, Secretaria de Meio Ambiente, Infraestrutura e Logística do estado de São Paulo, Brasil; 5 P. B. Šivickis Laboratory of Parasitology, State Scientific Research Institute Nature Research Centre, Vilnius, Lithuania; Oswaldo Cruz Foundation: Fundacao Oswaldo Cruz, BRAZIL

## Abstract

**Background:**

*Trypanosoma cruzi* is a multi-host parasite that infects dozens of mammalian species in the most variable wild environments in Americas. Between 2013 and 2018, autochthonous infections by *T. cruzi* were suspected in three European wolves (*Canis lupus*) and an orange dwarf porcupine (*Coendou spinosus*) from *Fundação Parque Zoológico de São Paulo* (FPZSP), Brazil, current *Coordenadoria de Fauna Silvestre* (CFS), which is inserted in a remnant of the Atlantic rainforest inside one of the biggest and most populous municipality of the world. This study aims to detect *T. cruzi* infections in captive mammals, triatomines and free-living opossums from surrounding areas of FPZSP/CFS.

**Methodology/Principal Findings:**

Blood samples from captive and free-living mammals from surrounding areas were collected for parasitological (direct examination and culture), serological (IFAT) and molecular diagnosis using Nested-PCR *18SrDNA* followed by DNA sequence analysis. Triatomines (*Panstrongylus megistus*) found in FPZSP/CFS were also examined by culture of the digestive tract and PCR. *Trypanosoma cruzi* infection was detected in 35.7% (n = 60/168 – 106 captive and 62 free-living) of the mammals that belonged to nine different families. From captive mammals, positive *T. cruzi* serology was observed in 29.6% (n = 27/91). Twenty-six positive hemocultures were obtained, from which parasite isolation was achieved in 69.2%, while positive PCR was observed in 40% of them, including nine free-living individuals that were also positive in hemoculture. Of 28 individuals in which *T. cruzi* characterization was successful, 89.3% were genotyped as DTU TcI, 7.2% as TcII and 3.5% as TcI/TcII mixed infection. Besides, 29 of 30 collected triatomines were infected, and infection by *T. cruzi* DTU TcI was confirmed in 16 of them.

**Conclusions/Significance:**

The confirmed autochthonicity of at least 68.9% of cases demonstrates that captive mammals from FPZSP are immersed in the *T. cruzi* enzootic cycle that involves the vector species *P. megistus,* and the reservoir hosts *C. spinosus* and *Didelphis aurita* from the wild surrounding areas.

## Author summary

*Trypanosoma cruzi*, the causative agent of Chagas disease, is a parasite that infects multiple host species and is present in a wide range of environments. The *Fundação Parque Zoológico de São Paulo* (FPZSP), current *Coordenadoria de Fauna Silvestre* (CFS), is located in a large and preserved fragment of the Atlantic rainforest in one of the largest metropolis in the world. The premise of four *T. cruzi* autochthonous cases in captive animals led us to investigate the infection not only in captive fauna, but also in wildlife mammals and vectors in the surrounding area through parasitological, serological and molecular tests. Almost all examined kissing bugs, half of the captured *Didelphis aurita* opossums and approximately a quarter of the FPZSP/CFS captive animals tested were infected by *T. cruzi* and two distinct genotypes of the parasite (TcI and TcII) were identified. The autochthonous cases (animals born in captivity and with no history of displacement) and the high infection rates confirm local transmission of *T. cruzi*. The interaction between kissing bugs and infected free-living opossums from the surrounding areas with the FPZSP captive mammals is the main hypothesis that explains the infection in the zoo mammals.

## Introduction

*Trypanosoma cruzi* (Chagas, 1909) (Kinetoplastida; Trypanosomatidae) is a heteroxenous parasite of mammals and kissing bugs (Hemiptera; Triatominae), and the etiological agent of Chagas disease, a neglected tropical disease that affects about 6 million people worldwide resulting in 10,000 annual deaths [1]. Although mostly associated with human cases, *T. cruzi* is a native enzooty that circulate in South America millions of years before human arrival, first in the autochthonous fauna of marsupials and xenarthrans, and subsequently infecting other mammalian orders upon their arrival in the continent [2].

As one of the most ancient American parasite, *T. cruzi* is a successful multi-host parasite, being able to virtually infect all mammal species (and inside them, different cell types, excepting red blood cells) in different wild environments (including those modified by humans) from South Argentina to South United States and whose transmission can occur due to the vectorial contaminative route (infective stages is released by the vector during blood meal) or the oral route (by the ingestion of infected bugs or blood and/or tissues of infected animals) [3]. It is also a genetic diverse parasite that can be grouped into seven Discrete Typing Units (DTUs), named TcI to TcVI and Tcbat. The DTUs TcI to TcIV are widely dispersed in the wild, while TcV and TcVI are mostly associated with human cases, and Tcbat with bats [4]. Despite the efforts of different studies to associate the different DTUs to a certain species or order of mammal, ecotope or geographic distribution, no association has proved to be strict so far, especially considering the normally under sampling of these association proposals [5].

Captive animals are also exposed to *T. cruzi* infections in different enzootic scenarios. In the Brazilian Amazon, different captive primate species belonging to five families were exposed to the local *T. cruzi* transmission cycle, as demonstrated by blood positive PCR [6]. In the Brazilian Savannah, primates from Brasília Zoo that belonged to eight species were found infected by *T. cruzi,* including one *Callithrix geoffroyi* Humboldt, 1812 that became infected during the follow-up [7]. A few years later, a huge sampling effort in the same Zoo demonstrated *T. cruzi* infection in 24 species from Carnivora, Cetartiodactyla, Perissodactyla, Pilosa and Primates order, including ten infected individuals born at the Brasília Zoo [8].

Regarding the *T. cruzi* enzooty, the Atlantic Rainforest is one of the most studied biomes. In this environment it was described for the first time a stable TcII transmission in the golden-lion-tamarin *Leontopithecus rosalia* (Linnaeus, 1766) with *T. cruzi* infection being reported both in conserved and in very degraded areas, as is the case of the *Estação Biológica Fiocruz Mata Atlântica*, Rio de Janeiro (RJ), Brazil [9] and APA of *Bacia do Rio São João* (RJ) [10]. The *Fontes do Ipiranga* State Park (PEFI) is an Atlantic Rainforest fragment of 543ha that is in the Southeast region of the of São Paulo municipality, the biggest South American metropolis [11]. This fragment has four delimited areas: (i) a Biological Reserve, composed of Atlantic forested areas; (ii) the *Instituto de Pesquisas ambientais* – IPA; (iii) The *Fundação Parque Zoológico de São Paulo* (FPZSP), current *Coordenadoria de Fauna Silvestre* (CFS); (iv) the Science and Technology Park of USP (CienTec Park); and (v) some deforested areas [12].

The FPZSP/CFS and the adjacent Zoo Safari have an area of approximately 82 ha and are the home to more than 2,500 captive vertebrate animals, some born in the Zoo and others derived from the wild or other institutions. Many of its enclosures are surrounded by PEFI forest areas and wild mammals that can travel between the enclosures, especially during the night.

In 2013, the FPZSP/CFS veterinarians suspected of *T. cruzi* infection in a female of European wolf [*Canis lupus lupus* Linnaeus, 1758 (Carnivora, Canidae)] that presented cardiac alterations detected by clinical auscultation and electrocardiogram. Similar symptoms had been previously observed in her mated male, dead in 2010, and brought from Vancouver Zoo (Canada) in 1996, where *T. cruzi* infection is not reported in wild fauna. A retrospective study confirmed the *T. cruzi* infection by serology and PCR. In 2018, the infection was serologically confirmed in a third wolf, son of the Canadian couple and born in the FPZSP/CFS, with no displacement history. There was also suspicion of another autochthonous case from 2016, an orange dwarf porcupine [*Coendou spinosus* (Cuvier, 1822) (Rodentia; Erethizontidae)] whose blood contained flagellated trypanosomatids, observed by the buffy coat method [3]. A higher preoccupation emerged by the fact that some captive animals were reported to sporadically prey on free-living opossums [*Didelphis aurita* Wied-Neuwied, 1826 (Didelphimorphia: Didelphidae)], described as one of the most important reservoirs of *T. cruzi* [13].

This previous history led to the hypothesis that there was a local *T. cruzi* enzootic cycle involving other captive and free-living mammals, and triatomines from the surrounding forested areas. To test this hypothesis, we evaluated the occurrence of *T. cruzi* infection in captive mammals (*ex situ*) from the FPZSP/CFS, triatomines found inside the FPZSP/CFS and in small free-living mammals (*in situ*) in the surrounding area through serological, parasitological, and molecular assays.

## Material and methods

### Ethics Statement

The procedures of capture and sample collection was authorized by *Instituto Chico Mendes de Conservação da Biodiversidade*/ICMBio (SISBIO nº 58262–1) and São Paulo Zoo (52/2017/423). The study was conducted in accordance with the normative established by the Internal Biosafety Commission of Oswaldo Cruz Institute and approved by the Ethics Committee on Animal Use of Oswaldo Cruz Foundation (L-050/2016 and LA-009/2017).

### Study area

The study was conducted in the FPZSP/CFS (23°38’48“S, 46°37’48” W), a Brazilian Zoo that comprises an area of 82 ha located in the biggest Atlantic Forest fragment of São Paulo municipality, Brazil: The Fontes do Ipiranga State Park (PEFI). The climate is mild mesothermal (average between 10°C and 15°C), super humid and the PEFI’s vegetation is characterized by dense rain forest on the Atlantic hillside, in addition of anthropic fields and exotic trees. Mammals that live in the São Paulo Zoo are sheltered in enclosures inside the forest area or next to the edges of the forest or enclosures whose maximum distance from the edges is 500m.

### Captive wild mammals restraint and collect blood

**Fig 1:**
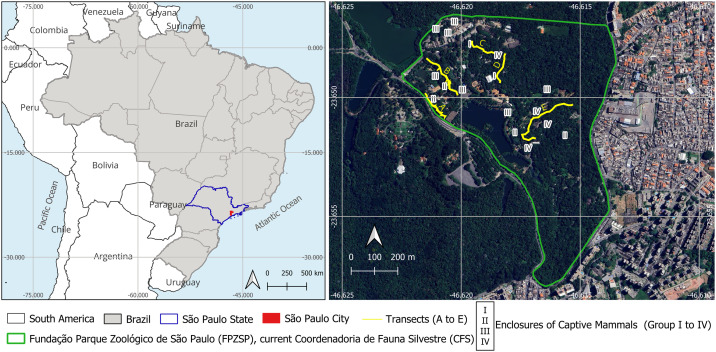
Study area located in The *Fundação Parque Zoológico de São Paulo* (FPZSP), current *Coordenadoria de Fauna Silvestre* (CFS). Study area located in São Paulo Zoo, municipality of São Paulo (São Paulo State/Brazil), showing the five transects (A to E) and some of the enclosures of captive mammals, according to the group they were included **(I to IV)**. This map was created using QGIS version 3.28.12 software (EPSG: 4076 - SIRGAS 2000) and cartographic bases maps modified from open access by the Brazilian Institute of Geography and Statistics, IBGE (https://www.ibge.gov.br/geociencias/downloads-geociencias.html).

Of the 451 mammals that were maintained in the FPZSP/CFS during the study, 106 were spitted into four groups, according to their putative exposition to the wild *T. cruzi* transmission cycle (Table 1 and Fig 1): Group I included mammals whose *Trypanosoma* sp. infection was previously detected; Group II included species reported to kill and/or prey free-living opossums; Group III included mammals whose enclosures are close to animals from Groups I and II; and Group IV included mammals whose enclosures are adjacent to the surrounding forest areas. Individuals were restrained for the first time (A) to collect blood and, after a minimum of four months for a second time (B), when possible. Physical and chemical restraints were performed according to routine protocols employed by the FPZSP/CFS Institution. The 106 individuals belonged to six orders, 15 families and 31 species (Table 1). Fifty-seven of them were submitted to the second sampling, in total 163 sampling events.

**Table 1:** Groups of captive wild mammals from the FPZSP/CFS for the two predicted samplings aiming the diagnosis of *Trypanosoma cruzi* infection.

Group	DESCRIPTION	MAMMAL			N Col. A	N Col. B
**Order/ Family**	**Scientific Name**	**Common Name**		
**I**	*Trypanosoma* sp. Infected species *(Index cases)*	Carnivora/ Canidae	*Canis lupus lupus*	Grey Wolf	1	1
		Rodentia/ Erethizontidae	*Coendou spinosus*	Porcupine	3	2
**II**	Mammals reported to prey/kill opossums (*Didelphis* spp.)	Carnivora/ Canidae	*Chrysocyon brachyurus*	Maned Wolf	4	4
		Carnivora/ Felidae	*Leopardus pardalis*	Ocelot	2	1
		Primates/ Cebidae	*Sapajus apella*	Black-capped Capuchin	12	10
		Primates/ Hominidae	*Pan troglodytes*	Chimpanzee	5	0
**III**	Mammals whose enclosures are located close to those from Groups I and II	Carnivora/ Canidae	*Cerdocyon thous*	Crab-eating Fox	1	0
			*Speothos venaticus*	Bush Dog	1	0
		Carnivora/ Felidae	*Leopardus geoffroyi*	Geoffroyi’s Cat	8	7
			*Leopardus wiedii*	Margay	2	1
			*Leopardus tigrinus*	Northern Tiger Cat	4	2
			*Leptailurus serval*	Serval	3	3
			*Leopardus braccatus*	Pampas Cat	2	2
			*Puma yagouaroundi*	Jaguarundi	3	2
		Carnivora/ Mustelidae	*Eira barbara*	Tayra	1	0
		Cetardiodactyla/ Camelidae	*Lama glama*	Guanaco	1	0
		Cetardiodactyla/ Tayassuidae	*Tayassu tajacu*	Collared Peccarie	1	1
		Didelphimorphia/ Didelphidae	*Didelphis aurita*	Opossum	1	1
		Pilosa/ Myrmecophagidae	*Myrmecophaga tridactyla*	Giant Anteater	1	0
			*Tamandua tetradactyla*	Southern Tamandua	1	0
		Primates/ Atelidae	*Ateles paniscus*	Guiana Spider Monkey	1	0
			*Brachyteles arachnoides*	Southern Muriqui	2	0
		Primates/ Cebidae	*Sapajus flavius*	Blonde Capuchin	1	1
		Rodentia/ Dasyproctidae	*Dasyprocta azarae*	Agouti	1	1
**IV**	Mammals whose enclosures are inserted or nearby the forested areas	Carnivora/ Canidae	*Lycaon pictus*	African Wild Dog	2	1
		Carnivora/ Felidae	*Panthera leo*	Lion	5	0
			*Panthera tigris tigris*	Tiger	3	1
		Carnivora/ Herpestidae	*Suricata suricatta*	Meerkat	4	0
		Carnivora/ Ursidae	*Ursus arctos*	Brown Bear	1	1
		Primates/ Atelidae	*Ateles* sp.	Sipder Monkey	1	0
		Primates/ Callitrichidae	*Leontopithecus chrysomelas*	Golden-headed Lion Tamarin	19	12
			*Leontopithecus chrysopygus*	Black Lion Tamarin	9	3
**TOTAL**					106	57

*N: Number of examined mammals. Col.: Collect (A: first sample collection; B: second sample collection).*

### Small free-living mammals capture

Small free-living mammals were captured in July 2017 (cold and dry season) and February 2018 (hot and wet season). We used 75 live traps, alternating Tomahawk (Tomahawk, WI, USA) and Sherman (H. B. Sherman Traps, Tallahassee, FL, USA). Live Traps were distributed in five transects of 10–25 trap points each. Traps were placed in the ground at 10m intervals during five consecutive nights, and baited with a mixture of banana, peanut butter, oat, and bacon or sardines [14]. The five transects were disposed next to the captive enclosures and/or next to the forest areas (Fig 1). The capture effort was 375 trap-nights per expedition (750 trap-nights in the two expeditions).

The identification of captured specimens was based on external morphological characters. The captured animals were anesthetized with an intramuscular injection (IM) of Ketamine (20–30 mg/kg) associated with Midazolam (0.05-0.1 mg/kg) and Fentanyl (0.02-0.05 mg/kg) for blood sample collection and individually identified with ear tags. At the end of the procedure, Flumazenil (0.01 mg/kg, IM) was administered. After anesthetic recovery, the animals were released at the same point of capture.

Additionally, eight free-living individuals were captured by the FPZSP/CFS team for different reasons: four opossums, two porcupines (*C. spinosus*), one brown howler monkey [*Alouatta guariba* (Humboldt, 1812) (Primates; Atelidae)] and one nine-banded armadillo [*Dasypus novemcinctus* (Lineu, 1758) (Cingulata; Dasypodidae)]. These animals were physically and chemically restrained through the routine protocols of the FPZSP/CFS, had blood samples collected and were also included in the study.

### Triatomines

Opportunistically, the triatomines found in the FPZSP/CFS facilities were manually captured. No active insect search methodology was carried out. The living bugs were placed in plastic collectors, with a perforated cover for air intake and with filter paper inside, while dead insects were placed in collectors containing 70% ethanol [15]. All insects were identified using dichotomous keys [16]. The intestinal contents from live individuals were examined on a microscope and cultured in Novy-Neal-Nicolle (NNN) medium with Liver Infusion Tryptose (LIT) overlay [14]. Intestinal contents from dead bugs were also collected and frozen for molecular analysis.

### Procedures with the blood samples

Blood samples were taken from both captive and wild mammals, this latter through cardiac puncture. The samples were used for parasitological and serological analysis, as follows: (i) the fresh buffy-coat [3] was obtained from the centrifugation of microcapillaries (15,800g/5 minutes) and the material placed between slide and coverslip; (ii) the hemoculture was performed by the inoculation of 0.6–0.8 mL of blood from each animal in two tubes containing NNN-LIT medium [17]; (iii) 0.3 mL of total blood was laid up with 0.6 mL of guanidine-EDTA [18] and held refrigerated; and (iv) the remaining blood was centrifuged (1.120g/5 minutes) and the serum stocked at −20°C. Priority was given to hemoculture when insufficient blood volume was obtained.

### Parasitological diagnosis, molecular characterization and phylogenetic tree

The fresh buffy coat was examined in the field lab using an optical microscopy at 400 × magnification [3]. Hemocultures were maintained in a BOD chamber at 27^o^C and evaluated every 15 days for up to five months. When positive, cultures were amplified, cryopreserved, and deposited in the Collection of *Trypanosoma* from Wild and Domestic Mammals and Vectors (COLTRYP/Fiocruz-RJ). The COLTRYP numbers of the sequences are listed in S1 Table.

Material derived from positive cultures and triatomine intestinal samples had their DNA extracted by the standard phenol-chloroform method [19]. DNA from total blood with guanidine-EDTA was extracted with DNeasy Blood & Tissue Kit (QIAGEN, EUA), according to manufacturer’s instructions.

The obtained DNAs were submitted to a Nested PCR targeting the *18SrDNA* gene for trypanosomatid diagnosis using two sets of primers previously described [20,21]: TRY R (5’CTACTGGGCAGCTTGGA3’) and F (5’GAAACAAGAAACACGGGAG3’) were used in the first round, and SSU R (5’CTGAGACTGTAACCTCAAAGC3’) and F (5’TGGGATAACAAAGGAGCA3’) in the second round. Both rounds were completed with 30 cycles, initial denaturation at 95^o^C for 3 minutes, followed by 30 cycles at 94^o^C for 30s, 55^o^C for 60s, 72^o^C for 90s, 72^o^C for 10 minutes and kept at 4°C. The PCR products electrophoresis was held on 2% agarose gel, stained with GelRed (Biotium, USA) and visualized under UV light. Products with approximately 600 bp of DNA were purified using GFX PCR DNA and Gel Band Purification Kit (GE HealthCare, UK) according to manufacturer’s instructions.

The Sanger sequencing reaction was performed for both strands using the BigDye Terminator v3.1 kit (Applied Biosystems, USA) at the Sequencing Platform of the Oswaldo Cruz Foundation/Rio da Janeiro - RJ. The obtained consensus sequences were manually edited using the SeqMan-DNA Star Program and the species were identified using the BLAST (Basic Local Alignment Search Tool) algorithm according to the similarity (minimal percent identity 99%; E-value 0.0; query coverage 100%) with reference sequences on GenBank (NCBI).

The alignment of the sequences was also obtained by BioEdit software, and the phylogenetic trees were built with MEGA X software, using the Neighbor-Joining (NJ) methods (Kimura model 2 parameters with 1000 bootstraps) and Maximum Likelihood (ML), according to the best model for each group of sequences, with the lowest Bayesian Information Criterion (BIC). The access numbers to the GenBank of the reference sequences are listed in S2 Table.

### Serological tests

A serological survey for anti-*T. cruzi* IgG detection was performed using an adapted version of the Indirect Immunofluorescent Antibody Test (IFAT) described by [22]. Antigens used in the reaction were mixed in equal proportions of complete parasites from reference strains of *T. cruzi* DTU TcI (I00/BR/00F) and DTU TcII (MHOM/BR/1957/Y).

The reaction was revealed by fluorescein-conjugated antibody according to phylogenetic proximity to the investigated species: primates were tested with anti-human IgG conjugate (Bio-Manguinhos); canids and felids were screened with commercial conjugates (FITC), anti-dog IgG and anti-cat IgG respectively; and *Didelphis*’ reactions were revealed by a commercial anti-rabbit IgG conjugate (FITC) and *in house* specific intermediary anti-*Didelphis* antibodies raised in rabbits [9]. All reactions included positive and negative serum samples from the same host species of the employed conjugate. The cut-off values adopted were 1:40 for *D. aurita*, canids and felids; and 1:10 for primates, as previously described [2,23]. Other mammals were not tested due to the absence of available conjugates.

To diagnose cross-reactions with *Leishmania* spp., animals were also examined for the presence of anti-*Leishmania* sp. IgG by IFAT using antigens from a mixture of *Leishmania braziliensis* Vianna, 1911 (MHOM/BR/1975/M2903 – IOC/L566) and *L. infantum* Nicolle, 1908 (MHOM/BR/1974/PP75 – IOC/L579) parasites, derived from *Leishmania* Collection from Oswaldo Cruz Institute (CLIOC/Fiocruz-RJ). The same conjugates and conditions employed for anti-*T. cruzi* IFAT was employed. Canidae were also evaluated by a rapid test of Canine Visceral Leishmaniasis (CVL/TR-DPP), as described elsewhere [24].

Whenever the result was the exact cut-off value for both *T. cruzi* and *Leishmania* spp., it was considered indeterminate. Besides, if the titration of anti-*T. cruzi* was the exact cut-off value and of anti-*Leishmania* sp. was higher or the molecular characterization detected infection by other trypanosomatid, we consider that a cross-reaction occurred, and these animals were not considered infected by *T. cruzi*. Animals that presented IFAT titters at least one titration above the cut-off value were considered infected by *T. cruzi*.

### Ecological network and statistical analysis

A matrix was created to reproduce the interaction between captive mammals and *T. cruzi* infections. In this matrix (a), mammals were represented in lines (i), *T. cruzi* in one column (j) and the interactions between them were produced by the elements a_ij_ [25]. The quantitative matrix was elaborated considering how many times the parasite was diagnosed in each mammal species. From that matrix, a two-mode network of mammal-parasite interactions was constructed. This matrix was only about mammal-*T. cruzi* interactions, without considering interactions from mammal-mammal. The two-mode network was created using the ‘bipartite’ [26], ‘sna’ [27] and ‘statnet.common’ [28] packages at R software [29]. Statistical analyzes were performed using Pearson’s chi-square test, adopting a significant level of 0.5% (p < 0.005).

### Map construction

The points of capture were georeferenced through the Global Positioning System receiver (GPS eTrex Garmin), with World Geodetic System 84 (WGS 84) as the geodetic reference. The study area and the transects were visualized in Quantum GIS software version 3.28.14, with municipal boundaries extracted from the base of IBGE and with Google Earth Satellite images (QGIS QuickMapServices plugin).

## Results

From the 168 examined animals, 106 captive (*ex situ*) and 62 free-living (*in situ*), *T. cruzi* infection was detected in 35.7% (n = 60/168) of them, which belonged to nine different families. Despite being apparently higher, the infection rate among free-living (50%; n = 31/62) did not statistically differ (*x*^*2*^ = 0.79685; df = 1; p < 0.005) than among captive mammals (27.3%; n = 29/106). Among the 60 infected individuals, except for two that were not submitted to the IFAT, the serological infection rate was 83% (49/58). From 26 positive blood cultures (43.3%; n = 26/60), parasites were isolated in 69.2% (n = 18/26), being possible to characterize 13 of them. Besides, the PCR analysis performed in the whole blood with guanidine-EDTA detected the infection in 40% (n = 24/60) of the sample, including nine free-living mammals that were also positive in the hemoculture. From 28 animals in which *T. cruzi* characterization was successful, 89.3% (n = 25/28) were genotyped as DTU TcI, 7.2% (n = 2/28) as DTU TcII and one sample (3.5%; n = 1/28) as TcI/TcII mixed infection (LBT 10257). Besides, 29 of 30 collected triatomines [*Panstrongylus megistus* [Burmeister, 1835]] were infected, of which 55.1% (n = 16/29) were confirmed as *T. cruzi* DTU TcI.

### Mammals *ex situ*

Three golden-headed lion tamarins [*Leontopithecus chrysomelas* [Kuhl, 1820]] presented positive results in the fresh buffy-coat examination (2.8%; n = 3/105). At least in one of them, the tripomastigote forms were exceptionally large, resembling *Trypanosoma minasense* Chagas, 1908, which was further confirmed in the molecular diagnosis in blood. Five hemocultures (4.7%; n = 5/105) derived from *ex situ* mammals were positive. From these, two were established and it was possible to identify *T. cruzi* DTU TcI in one opossum and one golden-headed lion tamarin, which was also positive in fresh buffy-coat examination (Table 2).

**Table 2:** *Trypanosoma cruzi* infection detected by parasitological (hemoculture and PCR) and serological (IFAT) assays in captive wild mammals from the FPZSP/CFS, Brazil.

Group	Mammals			Molecular Characterization A and B	IFAT: A and B Pos. (+), Neg. or ND	Infection Rate
	Scientific Name	Pos./Total				
**I**	*Canis lupus lupus*	1/1			A + /B+ (n = 1)	50% (2/4)
	*Coendou spinosus*	1/3		Blood PCR: *T. cruzi *DTU TcI (B n = 1)	A ND/B ND	
**II**	*Leopardus pardalis*	1/2			A + /B+ (n = 1)	26.1% (6/23)
	*Sapajus apella*	2/12		Blood PCR: *T. cruzi* DTU TcI (B n = 1)	A Neg./B+ (n = 2)	
	*Pan troglodytes*	3/5			A + /B ND (n = 3)	
**III**	*Cerdocyon thous*	1/1			A + /B ND (n = 1)	22.8% (8/35)
	*Leopardus geoffroyi*	5/8			A + /B Neg. (n = 2) A + /B ND (n = 1) A + /B+ (n = 2)	
	*Leptailurus serval*	1/3			A + /B+ (n = 1)	
	*Didelphis aurita*	1/1		Hemoculture: *T. cruzi *DTU TcI (B n = 1)	A + /B+ (n = 1)	
**IV**	*Lycaon pictus*	2/3			A + /B+ (n = 1) A + /B ND (n = 1)	29.5% (13/44)
	*Panthera leo*	1/5			A + /B ND (n = 1)	
	*Panthera tigris tigris*	3/3		Blood PCR: *T. cruzi* DTU TcI (A n = 1)	A + /B ND (n = 2) A + /B+ (n = 1)	
	*Leontopithecus chrysomelas*	7/19		Hemoculture: *T. cruzi* DTU TcI (A n = 1) Blood PCR: *T. cruzi* DTU TcI (A n = 1; B n = 3)	A + /B+ (n = 3) A + /B ND (n = 3) A ND/B ND (n = 1)	
**TOTAL *T. cruzi***			**9 *T. cruzi* DTU TcI** (Hemoculture = 2; Blood PCR = 7)		**27**	**27.3%** (29/106)

*A: first collect. B: second collect. Pos.: positive. Neg.: Negative. ND: not done. PCR: Polymerase Chain Reaction; IFAT: Indirect Fluorescent Antibody Test*

The molecular diagnosis in total blood was performed for all 106 mammals, of which seven (6.6%) were infected with *T. cruzi* DTU TcI (Table 2). Among these, there are included one *L. chrysomelas* positive in the fresh buffy-coat examination and one also in hemoculture. Overall, the molecular characterization in blood and hemocultures demonstrated the infection by *T. cruzi* DTU TcI in nine captive mammals (8.5%; n = 9/106) (Table 2).

Of the 91 individuals in which the IFAT was performed, 29.6% (n = 27/91) were infected by *T. cruzi* (Table 2). Two seroconversion events were observed in two capuchins (*Sapajus apella* Linnaeus, 1758), one of them also confirmed by total blood PCR. One Geoffroyi’s cat (*Leopardus geoffroyi* d’Orbigny & Gervais, 1844] presented a possible cross-reaction, once the titration of anti-*T. cruzi* was at cut-off value and the molecular diagnosis identified infection by *Trypanosoma rangeli* Tejera, 1920. Two primates (a chimpanzee and a spider monkey) showed undetermined results and all other animals were negative for *Leishmania* sp. infection.

Considering all diagnostic tests, the infection rate by *T. cruzi* in captive mammals was 27.3% (n = 29/106) and among these 68.9% (n = 20/29) are proven autochthonous, since 18 of these positive animals were born in the FPZSP/CFS and two derived from the surrounding PEFI. Considering the evaluated groups (II to IV), all of them had at least one individual infected by *T. cruzi* and the infection rates were not statistically different (*x*^*2*^ = 0.9987; df = 2; p < 0.005).

**Fig 2:**
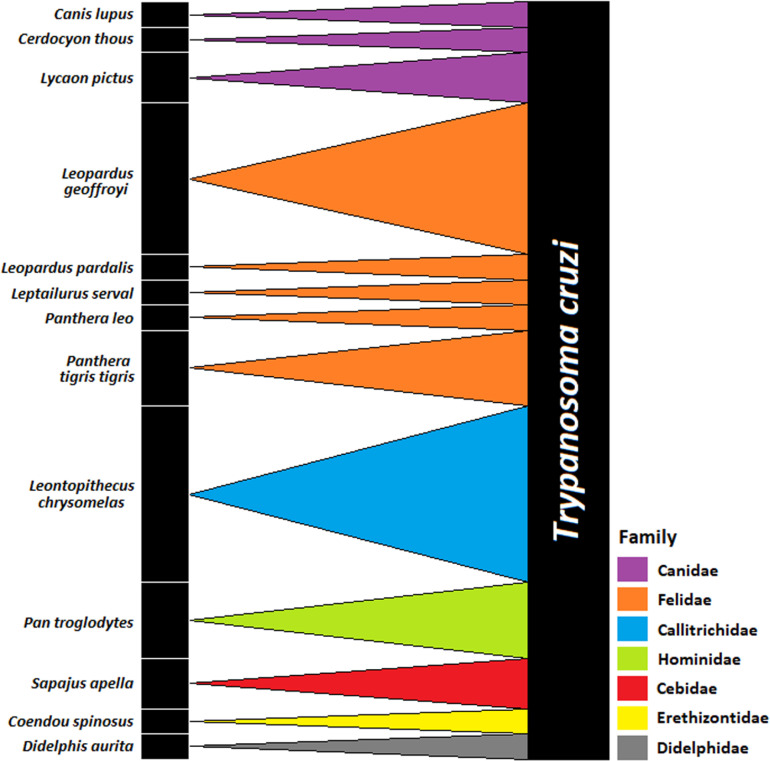
Quantitative ecological network among captive wild mammals from the FPZSP/CFS and *Trypanosoma cruzi.* The bars on the left represent the infected mammals’ species and on the right the protozoan. The frequency of interaction between each species and the protozoan is illustrated by the bars’ length. The links represent the present interactions, and the thickness is proportional to their frequency, represented by distinct colors for each mammalian family.

There are infected animals in five families from two orders (Canidae and Felidae, from Carnivora; Callitrichidae, Cebidae and Hominidae, from Primates). Despite the infection rate among Carnivora (31.9%; n = 15/47) seems to be higher than Primates (24%; n = 12/50), no statistical difference was observed (*x*^*2*^ = 0.9381; df = 1; p < 0.005). Of all infected animals 37.9% (n = 11/29) belong to the family Felidae, whose infection rate was 34.3% (n = 11/32), being the highest rate among all studied families, followed by Callitrichidae (25%; n = 7/28), although without statistical difference (*x*^*2*^ = 0.9241; df = 1; p < 0.005) (Fig 2).

### Mammals *in situ*

Overall, 54 opossums (*D. aurita*) were captured in live traps, resulting in a capture success of 7%. Added to the other eight individuals captured by the São Paulo Zoo team, a total of 62 free-living animals were examined. Considering all the diagnostic assays, the infection rate by *T. cruzi* was 50% (n = 31/62): 30 opossums and one porcupine.

**Fig 3:**
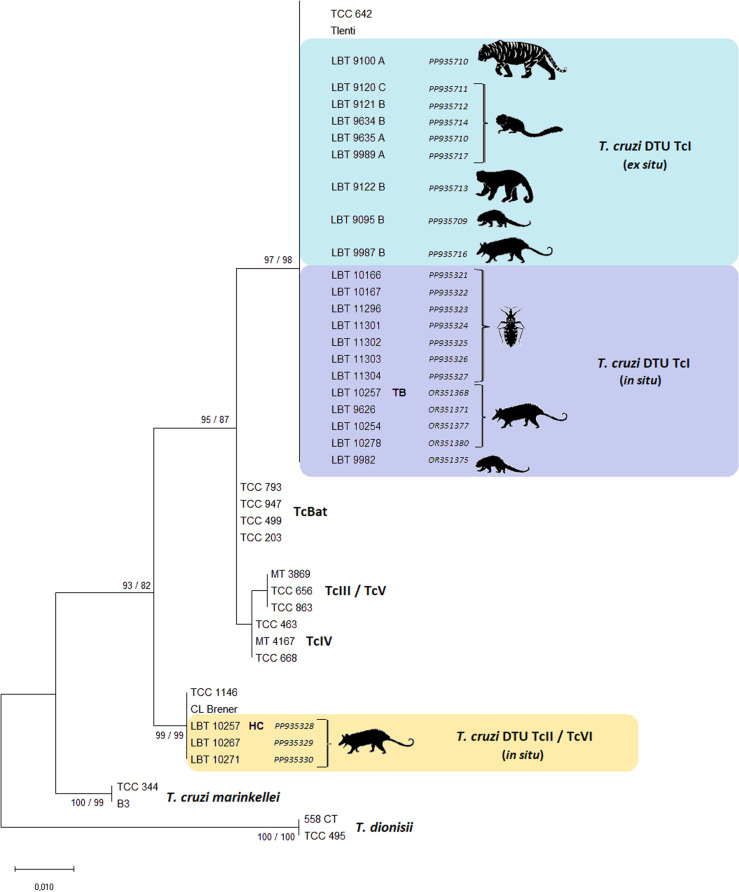
Phylogenetic tree reconstructed from sequences of the *18S SSU rDNA* gene of parasites of the genus *Trypanosoma* belonging to the *T. cruzi* clade. Data originated from: nine captive mammals from the FPZSP/CFS (*ex situ*), seven free-living mammals (*in situ*) and seven triatomines. Forty-two nucleotide sequences were used in the analysis, 18 of which were previously published. The numbers include the statistical support values of the inferences, respectively, by the Neighbor-Joining (NJ) (Kimura’s 2-parameter model) and Maximum Likelihood (ML) (Kimura’s 2-parameter model and four-category).

Both fresh buffy-coat examination and blood cultures presented more than 25% of positivity (26.6% and 33.8%, respectively), all from opossums. Of the 21 positive hemocultures, 16 resulted in isolates and 11 were successfully characterized as *T. cruzi* (eight DTU TcI and three DTU TcII). Likewise, the diagnosis in total blood with guanidine-EDTA identified 28.3% (n = 17/60) of positivity by *T. cruzi* DTU TcI: 16 opossums and one porcupine. Therefore, 45.2% of free-living individuals (n = 28/62) were positive in parasitological assays (fresh buffy coat, hemoculture and/or blood PCR) and it was possible to genotype the parasite in 19 of them: 16 DTU TcI, two DTU TcII and one mixed infection in one opossum (LBT 10257): TcI (total blood = TB) and TcII (hemoculture = HC) (Fig 3).

The IFAT was carried out for 57 opossums and one brown howler monkey, with 38% (n = 22/58), all opossums, being positive. It was not possible to evaluate the seroconversion in opossums, because even with the individual identification by ear tags, no animals from the first period were recaptured during the second period.

### Triatomines

Thirty adults *P. megistus* were examined, of which 29 (96.7%) were captured inside or near the porcupine enclosure and one inside the enclosure of the African wild dog [*Lycaon pictus* (Temminck, 1820)]. Twenty-seven were examined alive, and 26 (96.3%; n = 26/27) of them presented flagellates in the fresh examination of intestinal content. Twenty-four positive hemocultures (88.8%; n = 24/27) were obtained, including the only sample that was negative in the fresh examination, and 15 of them were characterized, all as *T. cruzi* DTU TcI. The molecular analysis performed in the frozen digestive tract of the three dead bugs demonstrated the infection in two of them, one characterized as *T. cruzi* DTU TcI. Accordingly, the triatomines’ infection rate was 97% (n = 29/30), with 55.1% (n = 16/29) confirmed as *T. cruzi* DTU TcI (Fig 3). Infection by *T. cruzi* was observed in both males and females and most of the insects examined were adults, besides few fourth and fifth instar nymphs.

## Discussion

The *T. cruzi* infection rate was high and above 25% among all studied groups from the FPZSP/CFS and surroundings: 27.3% for captive individuals, 50% for free-living animals (almost all opossums), and 97% for the collected *Panstrongylus megistus* triatomines. Besides the high rates of *T. cruzi* infection in opossums and bugs in the surrounding area, the confirmed autochthonicity of at least 68.9% of cases among the captive mammals confirmed the existence of a local transmission cycle of *T. cruzi* driven by the intrinsic local ecological interactions. The local *T. cruzi* transmission among captive mammals inside a Brazilian Zoo was already reported in Brasília, federal district [7,8], indicating that this phenomenon may be even broader, especially in Zoos maintained in very close proximity, even inside, pristine areas. Moreover, this study also provides the first description of *T. cruzi* infection in *C. l. lupus, C. spinosus, Leptailurus serval* (Schreber, 1776)*, L. pictus, Panthera leo* (Linnaeus, 1758), and *Panthera tigris tigris* (Linnaeus, 1758).

We could observe an elevated frequency of high parasitemia in opossums (43.1%; 25/58), demonstrated by positive hemocultures and/or buffy-coat fresh examinations. These results highlight the infective potential of opossums when preyed by other mammals, including the captive ones, and as source of infective blood meal for vectors. Surprisingly, eight opossums positive in fresh blood examination, hemoculture and/or PCR, were seronegative. The high rate of animals displaying high parasitemia, expressed by positive fresh blood examination or hemoculture, may be characteristic of the initial phase of infection. One possible explanation for the negative serology is the immunological window, a period in which the animal has not yet produced sufficient antibodies to be detected in the serological reaction, which would corroborate the hypothesis of recent infections. The fresh examination of intestinal triatomines content also showed a high rate of infection (96.2%; 26/27), highlighting the well-established transmission cycle of *T. cruzi* in the area.

Except for Group I, composed of mammals whose infection by *Trypanosoma* sp. was previously reported, the highest *T. cruzi* infection rate among captive mammals (29.5%) was diagnosed in Group IV, whose enclosures are adjacent to the forested surrounding area. However, the absence of statistical difference in comparison to other groups (Groups II and III) demonstrates that all factors considered to differ these three groups are important for mammal’s exposure to *T. cruzi* infection: (i) proximity to the area of forest, (ii) history of opossums’ predation, and (iii) proximity to mammals known to be infected by trypanosomatids and/or to mammals that prey opossums. Regarding the “proximity to the forest” factor, it should be noted that of the captured infected opossums, 75% were on transects located on the forest (50% on transect E and 25% on transect D), at a maximum distance of 150 m from the enclosures of all the evaluated captive animals. This distance is extremely short both for a *D. aurita*, whose living range can be up to 27 km^2^ [30], and for a *D. albiventris*, whose daily displacement can reach distances of up to 2.9 km [31]. Besides, all infected triatomines were found in enclosures next to the surrounding forest. Twenty-eight of the 29 infected triatomines were in or around the infected porcupine enclosure. The number of insects collected in a single enclosure points out to be a possible hotspot for the maintenance of these insects and their association with a given host. This was the same scenario observed in the Brasília Zoo, where a colony of 19 *P. megistus*, five of them infected, was detected in the porcupine enclosure [8]. It is worth also mentioning that there was no light nearby that was turned on at night and the closest light was from a conventional streetlamp, whose bulb is approximately 8–10 m high. This streetlight is not sufficient to illuminate the enclosure and, therefore, we discard the light attraction to explain the presence of *P. megistus* in this enclosure. In the FPZSP/CFS, the porcupine’s enclosure is about 10 m from the enclosure of the infected captive *D. aurita* and 110 m from the enclosure of the golden-headed lion tamarins (*L. chrysomelas*), whose infection rate was 36.8%. The other infected triatomine was found in the enclosure of the wild African dogs (*L. pictus*), which was serologically positive for *T. cruzi* infection. Studies on the dispersion of triatomines show that vectors could displace over distances of 100 m, depending on the species [32,33]. Under experimental conditions, *P. megistus* can remain in stationary flight for a period longer than one hour [34], in addition to having a displacement of up to 400 m [35]. Therefore, the proximity between the forest and the enclosures possibly facilitates the encounter between infected vectors and opossums with captive mammals, resulting in their infection.

The overall *T. cruzi* infection observed in the FPZSP/CFS was 27.3% among the captive wild mammals and 96.7% among the collected triatomines. In the Brasília Zoo, Minuzzi-Souza *et al.* [7] also observed high *T. cruzi* infection rate (88%) in *P. megistus* triatomines (including both adults and nymphs), and 65.4% of primates infected by *T. cruzi,* much higher than observed for primates in FPZSP/CFS (24%). Some years later, Reis *et al.* [8], searched 63 sites for triatomine’s presence in the same Zoo, and triatomines were found in only one (the porcupine enclosure), and in low infection rate (31.2%). Despite that, the *T. cruzi* infection in wild captive mammals remained elevated (67.6%) in the Brasília Zoo [8].

The oral route is considered the most ancient and successful transmission route in nature [2]. From captive animals from Group II, which have a history of predation on opossums, infection by *T. cruzi* was found in six individuals from three different species. In addition, of the nine species of the order Carnivora from Group III, four presented infected individuals. Although the absence of opossum predation events by tigers and other top predators from Groups III or IV, it is known that these animals can prey on small and medium vertebrates, in addition to invertebrates. In fact, this was a common trait in all infected captive animals, except for the porcupine (*C. spinosus*). However, this trait does not prevent the porcupine from consuming it, because of sensory and exploratory behaviors [36]. It should be noted that captive mammals inhabit enclosures that allow contact with triatomines from the forest and sometimes also with free-living opossums, depending on the structure of the enclosure. The contaminative vector route may also occur in the FPZSP/CFS, although we believe it is less frequent than the oral route, also because the animals tend to lick the bite, ingesting the insect’s feces. In animals with a dense coat, which can make it difficult the contact between insect’s feces and bite site, the probability of transmission via the contaminative vector route also tends to be low [13].

Using the number of each interaction as parameter, it was possible to identify interaction links with different thicknesses: the greater the number of interactions, the greater the thickness of the links (Fig 2). Thus, we found a strong interaction of *T. cruzi* with *L. geoffroyi* and *L. chrysomelas*, possibly due to, respectively, opossum and vector predation and proximity of the porcupine enclosure (<100 m). About the opossums’ predation, it is important to emphasize that PEFI is a very degraded forest fragment, which not only favors the contact between captive mammals and opossums but also favored our 7% capture success. Regarding the proximity of the porcupine enclosure, we emphasize its interaction with *P. megistus*, reported as the main vector in São Paulo state [37]. As 29 of the 30 insects were found in the porcupine’s enclosure (one of which was feeding in the moment that it was collected), we searched for colonies in trunks and crevices. However, no colonies were found, which made us conclude that the insects come from the surrounding forest, where they can live in different environments, such as palm trees, tree holes, bromeliads, shelters for terrestrial and flying mammals, bird nests, as well as houses and peridomestic environments (such as chicken coops) [38].

The different diagnostic techniques used, and the approach employed to evaluate two times some of the captive mammals proved to be complementary and enabled us to detect these high rates of infection. The different parasitological and molecular assays employed, as well as the possibility of a second sampling in half of the captive mammals, allowed us to detect different *T. cruzi* genotypes, including one mixed infection (TcI and TcII) in an opossum. Concomitant infections with different *T. cruzi* genotypes are common in nature, especially in marsupials, being the association TcI/TcII the most common in the Brazilian Atlantic Forest biome [13]. In our study, the *T. cruzi* DTU TcII population grew and was established in hemoculture, probably suppressing the growth of the DTU TcI population, which should also be present in the culture, but was diagnosed only in total blood PCR. It should be noted that the mixed infection between different *T. cruzi* genotypes was found only in this opossum in the FPZSP/CFS, and that opossums are considered a bioaccumulator of *Trypanosoma* sp. and *T. cruzi* DTUs [39].

In the FPZSP/CFS scenario and considering the concept of a reservoir as an ecological system formed by the species that keep the parasite in a given area, in a certain period of time [13], we can state that the opossums *D. aurita* are reservoirs of *T. cruzi* in the surrounding areas of the São Paulo Zoo, and this role is played by the captive porcupine inside the São Paulo Zoo. Although rodents were already described as having a secondary role as a reservoir in nature, the scenario can be different depending on the species or biome as is the case of *Rattus rattus* Linnaeus, 1758 and *Thrichomys laurentius* Thomas, 1904 in the Caatinga [39,40]. Among the heterogeneous group of rodents, porcupines show characteristics like those found in potential reservoirs, such as adaptation to different ecological niches and wide distribution [13]. We evaluated five individuals of *C. spinosus* (three captives and two free-living), of which two (40%) were infected, one free-living (PEFI) and one captive, but probably born at PEFI. A third captive porcupine, but also from PEFI, died in 2017, between the first and second collections. The results of the diagnoses performed with the samples from the first collection were negative, but the histopathological examination performed in the dead animal showed spleen and pulmonary parasitism. This animal used to live in the same enclosure of the infected captive porcupine, who had flagellated trypanosomatids in the blood since 2016. A unique aspect of this species is that the coat, being composed of a mixture of cylindrical aculeiform guard hairs and thin and long overcoats, can facilitate the blood meal since it can hinder the removal of insects by the porcupines [41]. This barely covers the skin surface of the porcupine, facilitating the contact of the feces with the bite site [42].

The pathogenesis involved in *T. cruzi* infection in wild mammals, even those maintained in captivity, is still poorly described. It is known that some of the nonspecific acute clinical signs may go unnoticed, but cardiac alterations detected by electro and echocardiograms, and the presence of myocarditis have been observed in nonhuman primates [43–47] and in a polar bear (*Ursus maritimus*) from the Guadalajara Zoo in Mexico [48]. In the animals from FPZSP/CSF, except for the wolves that were considered index cases for the present study, cardiac alterations were diagnosed only in one *L. pictus* and one *L. chrysomelas*, but these alterations could not be directly related to infection by *T. cruzi*. The general health status of all the other animals examined mammals was considered good.

An interesting aspect about São Paulo Zoo scenario is the possibility of a long-term follow-up that is important to assess the infective potential of wild mammals, which is usually difficult in natural infections at the forest or even in experimental infections. Besides, laboratory conditions do not mimic the conditions of nature [39]. However, there are some limitations to this kind of study, in particular, the inclusion of animals to be investigated (including resamples), which depend on the handling dynamics of that Zoo for routine examinations or emergency procedures in these animals, since they were not captured solely for the purpose of this study. Thus, the intervals between collections vary and make long-term monitoring difficult. In addition, some animals could not be included because their handling at the time of the study was not recommended due to different health issues. The impact of *T. cruzi* infections in the wild captive fauna is also difficult to measure since the timing, route of infection and initial parasite load are not known. In addition, co-infections and the impact of non-transmissible diseases can modify the course of *T. cruzi* infection, resulting in greater or lesser parasitemia; and, therefore, impact its detection.

Despite these inherent limitations, wild captive animals can represent natural infections more reliably than laboratory animals. When animals are inserted into a natural environment, they are susceptible to environmental factors and ecological interactions more like those found in nature. Thus, zoos allow us to expand our knowledge about the possible impact of parasites on the health of different wild mammals and the infection patterns of the different diagnosed parasites. In this sense, it is important to establish measures to prevent *T. cruzi* transmission among captive animals, especially those who are part of conservation programs, as well as those that can be transferred to another institution in captivity or reintroduced into the wild. It is worth mentioning that movements can be the subject of legislation or strict controls that should include the impact of reducing disease transmission [48]. Prevention measures must be analyzed, considering the importance of conservation and biodiversity of the parasites in the FPZSP/CFS. Thus, two measures would be to remove some key species from open enclosures and install screens in them. These alternatives aim to reduce the encounter of captive mammals with small free-living mammals and/or triatomines. In addition, mammals whose enclosures have trunks and wooden boxes (used as a burrow) must be constantly monitored in search of triatomine colonies. In addition, it is essential to carry out the clinical follow-up of infected captive mammals, as well as to adopt measures to prevent accidental transmission of these animals to São Paulo Zoo employees, such as the use of gloves when handling the animals, especially when in contact with animal’s blood. Drawing or manipulation of biological samples during surgical and dental procedures, necropsies, or even needles accidents are usually involved in most of the reported accidents [7].

It is worth remembering that the *T. cruzi* enzootic cycle does not only occur in the São Paulo Zoo, but it is part of nature and the Atlantic Forest [39,49], with different distribution patterns throughout Brazil, including probably also the unsampled areas. We consider the risk to humans to be extremely low, as nobody sleeps at the Zoo, even considering visits at night, because bugs are large and easily detected. Besides, both places commonly visited and the food storage for animals are always monitored by Zoo staff. Furthermore, zoo animals and local free-living animals could serve as blood sources for the vectors, maintaining the transmission cycle as an enzooty. This does not mean that monitoring should not be constant and considered by the surveillance authorities of the São Paulo municipality, especially because there are reports of *Trypanosoma* infected *P. megistus* in urban areas of the metropolitan region of São Paulo, including one infected insect captured in the Jabaquara subway, around 2km from the PEFI [50,51]. All possible measures to prevent the contact between humans and triatomines are important to avoid *T. cruzi* transmission.

*Trypanosoma cruzi* is a parasite that deserves to be highlighted within the context of One Health, as it has a complex transmission cycle, involving several hosts and vector transmission. In addition, the parasite has already been diagnosed in different ecological scenarios, from preserved to degraded environments. Environmental changes are altering the way parasites influence not only wildlife but entire ecosystems as well. Studies carried out in natural environments and captive institutions seeking to monitor and understand the relationship of parasitism with a focus on One Health are essential to advance and strengthen knowledge and conservation of biodiversity.

## Supporting information

S1 Table COLTRYP number of sequences obtained in this study (*18S SSU rDNA*). Bolded sequences are presented in Fig 3.
(DOCX)

S2 Table Sequences retrieved from GenBank and obtained in this study (highlighted in bold) that were included in the phylogenetic analyses (*18S SSU rDNA*). (DOCX)
