# Supplementary material for: Trypanosoma cruzi (Chagas, 1909) transmission among captive wild mammals, triatomines and free-living opossums from surrounding areas in the São Paulo Zoological Park, Brazil
Source: PLoS Negl Trop Dis. 2025 Apr 28;19(4):e0013055. doi: 10.1371/journal.pntd.0013055 (PMC12101743; doi:10.1371/journal.pntd.0013055)
Supplement: S1 Table — Bolded sequences are presented in Fig 3. (DOCX) [file pntd.0013055.s001.docx]

**Table S1.** COLTRYP number of sequences obtained in this study (18S SSU rDNA). Bolded sequences are presented in Figure 3.

| **Isolate** | **Genotype** | **Species** | **COLTRYP** |
| --- | --- | --- | --- |
| ***T. cruzi*** | | | |
| **LBT 9989 A** | **TcI** | ***Leontopithecus chrysomelas*** | **C00789** |
| **LBT 10167** | **TcI** | ***Panstrongylus megistus*** | **C00791** |
| **LBT 11296** | **TcI** | ***Panstrongylus megistus*** | **C00859** |
| **LBT 11301** | **TcI** | ***Panstrongylus megistus*** | **C00837** |
| **LBT 11302** | **TcI** | ***Panstrongylus megistus*** | **C00838** |
| **LBT 11303** | **TcI** | ***Panstrongylus megistus*** | **C00843** |
| **LBT 11304** | **TcI** | ***Panstrongylus megistus*** | **C00841** |
| LBT 10264 | TcI | *Didelphis aurita* | C00794 |
| LBT 10053 | TcI | *Didelphis aurita* | C00786 |
| LBT 9987 B | TcI | *Didelphis aurita* | C00813 |
| LBT 9624 | TcI | *Didelphis aurita* | C00772 |
| LBT 9628 | TcI | *Didelphis aurita* | C00766 |
| LBT 9620 | TcI | *Didelphis aurita* | C00771 |
| LBT 10280 | TcI | *Didelphis aurita* | C00806 |
| LBT 10283 | TcI | *Didelphis aurita* | C00799 |
| LBT 10268 | TcI | *Didelphis aurita* | C00795 |
| LBT 11001 | TcI | *Panstrongylus megistus* | C00853 |
| LBT 11274 | TcI | *Panstrongylus megistus* | C00865 |
| LBT 11291 | TcI | *Panstrongylus megistus* | C00851 |
| LBT 11292 | TcI | *Panstrongylus megistus* | C00854 |
| LBT 11293 | TcI | *Panstrongylus megistus* | C00864 |
| LBT 11294 | TcI | *Panstrongylus megistus* | C00855 |
| LBT 11305 | TcI | *Panstrongylus megistus* | C00839 |
| LBT 9839 | TcI | *Panstrongylus megistus* | C00775 |
| LBT 9984 | TcI | *Panstrongylus megistus* | C00783 |
| **LBT 10257 HC** | **TcII** | ***Didelphis aurita*** | **C00796** |
| **LBT 10267** | **TcII** | ***Didelphis aurita*** | **C00798** |
| **LBT 10271** | **TcII** | ***Didelphis aurita*** | **C00807** |
