# Supplementary material for: Trypanosoma cruzi (Chagas, 1909) transmission among captive wild mammals, triatomines and free-living opossums from surrounding areas in the São Paulo Zoological Park, Brazil
Source: PLoS Negl Trop Dis. 2025 Apr 28;19(4):e0013055. doi: 10.1371/journal.pntd.0013055 (PMC12101743; doi:10.1371/journal.pntd.0013055)
Supplement: S2 Table — (DOCX) [file pntd.0013055.s002.docx]

**S2 Table.** Sequences retrieved from GenBank and sequences obtained in this study (highlighted in bold) that were included in the phylogenetic analyses (18S SSU rDNA).

| **Isolate** | **COLTRYP** | **Genotype** | **Acession Number GenBank 18S** |
| --- | --- | --- | --- |
| ***T. cruzi*** | | | |
| TCC 642 | - | TcI | FJ001624 |
| Tlenti | - | TcI | MH059788 |
| TCC 1146 | - | TcII | FJ001629 |
| MT 3869 | - | TcIII | AF303660 |
| TCC 863 | - | TcIII | FJ549376 |
| MT 4167 | - | TcIV | AF288661 |
| TCC 668 | - | TcIV | FJ183396 |
| TCC 463 | - | TcIV | EU755224 |
| TCC 656 | - | TcV | FJ183395 |
| CL Brener | - | TcVI | AF245383 |
| TCC 947 | - | TcBat | FJ001626 |
| TCC 793 | - | TcBat | FJ001634 |
| TCC 499 | - | TcBat | FJ001622 |
| TCC 203 | - | TcBat | FJ001617 |
| **LBT 9100 A** | - | **TcI** | **PP935710** |
| **LBT 9120 C** | - | **TcI** | **PP935711** |
| **LBT 9121 B** | - | **TcI** | **PP935712** |
| **LBT 9634 B** | - | **TcI** | **PP935714** |
| **LBT 9635 A** | - | **TcI** | **PP935710** |
| **LBT 9989 A** | **C00789** | **TcI** | **PP935717** |
| **LBT 9122 B** | - | **TcI** | **PP935713** |
| **LBT 9095 B** | - | **TcI** | **PP935709** |
| **LBT 9987 B** | - | **TcI** | **PP935716** |
| **LBT 10166** | - | **TcI** | **PP935321** |
| **LBT 10167** | **C00791** | **TcI** | **PP935322** |
| **LBT 11296** | **C00859** | **TcI** | **PP935323** |
| **LBT 11301** | **C00837** | **TcI** | **PP935324** |
| **LBT 11302** | **C00838** | **TcI** | **PP935325** |
| **LBT 11303** | **C00843** | **TcI** | **PP935326** |
| **LBT 11304** | **C00841** | **TcI** | **PP935327** |
| **LBT 9982** | - | **TcI** | **OR351375** |
| **LBT 9626** | - | **TcI** | **OR351371** |
| **LBT 10254** | - | **TcI** | **OR351377** |
| **LBT 10278** | - | **TcI** | **OR351380** |
| **LBT 10257 TB** | - | **TcI** | **OR351368** |
| **LBT 10257 HC** | **C00796** | **TcII** | **PP935328** |
| **LBT 10267** | **C00798** | **TcII** | **PP935329** |
| **LBT 10271** | **C00807** | **TcII** | **PP935330** |
| ***T. cruzi marinkellei*** | | | |
| TCC 344 | - | - | FJ001664 |
| B3 | - | - | FJ649484 |
| ***T. dionisii*** | | | |
| 558 CT | - | - | KY689928 |
| TCC 495 | - | - | FJ001667 |
